# Machine learning approach to topological graph descriptors of graphene nanoribbons

**DOI:** 10.3389/fchem.2026.1750413

**Published:** 2026-03-12

**Authors:** K. Jyothish, S. Roy, Chandra Sekar Ponnusamy, K. B. Gayathri, J. Sahaya Vijay, Tony Augustine

**Affiliations:** 1 Department of Mathematics, School of Advanced Sciences, Vellore Institute of Technology, Vellore, India; 2 Department of Integrative Biology, School of Bio Sciences and Technology, Vellore Institute of Technology, Vellore, India

**Keywords:** degree based topological descriptors, graphene nanoribbons, machine learning, regression analysis, composite semiconducting index

## Abstract

Bottom-up syntheses of graphene nanoribbons have gathered considerable research interest because of their electronic properties and quantum behaviors which enhance their significance in nanotechnology. The advancement of material’s design methods and applications heavily depends on understanding how structural topology influences functional properties. This study analyzed valency based molecular descriptors of graphene nanoribbons with machine learning techniques. We have computed various valency based molecular descriptors of graphene nanoribbons and studied their predictive power using the logistic regression machine learning technique by describing its receiver operating characteristic curve to analyze the topological features of these graphene nanoribbons. These descriptors represent quantitative measurements of crucial structural features of graphene nanoribbons that directly affect material properties. This predictive framework enables researchers to design graphene nanoribbons with specific functionalities while advancing their knowledge about structure-property relationships in this material class. Molecular descriptors combined with machine learning methods demonstrate the potential to accelerate the discovery process and optimization of advanced nanomaterials.

## 1 Introduction

Graphene, a flat monolayer of $sp^{2}$ carbon atoms densely packed into a two dimensional honeycomb lattice of carbon atoms, is a fundamental part of carbon based materials. Graphene’s mechanical characteristics, chemical reactivity, and potential for use in many applications have positioned it as a promising chemical material in the last few decades. The transformation of graphene into a semiconductor has attracted quite a lot of attention in the previous few years, as the presence of a substantial bandgap in graphene might further open up an incredible potential of this material for a larger number of applications. Recent advancements in the liquid phase exfoliation process and graphene field effect transistors were developed to improve the capabilities of scale-up and operations. Also, studies moved to the applications of flexible electronics, photodetectors, and advanced computational modelling bringing arguably the greatest graphene prospects in semiconductor and optoelectronics devices (Chae and Lee, 2014; Lu et al., 2013; Nguyen and Nguyen, 2016; Shin et al., 2016; Sur, 2012; Yang et al., 2008; Kumar and Pattammattel, 2017).

**FIGURE 1:**
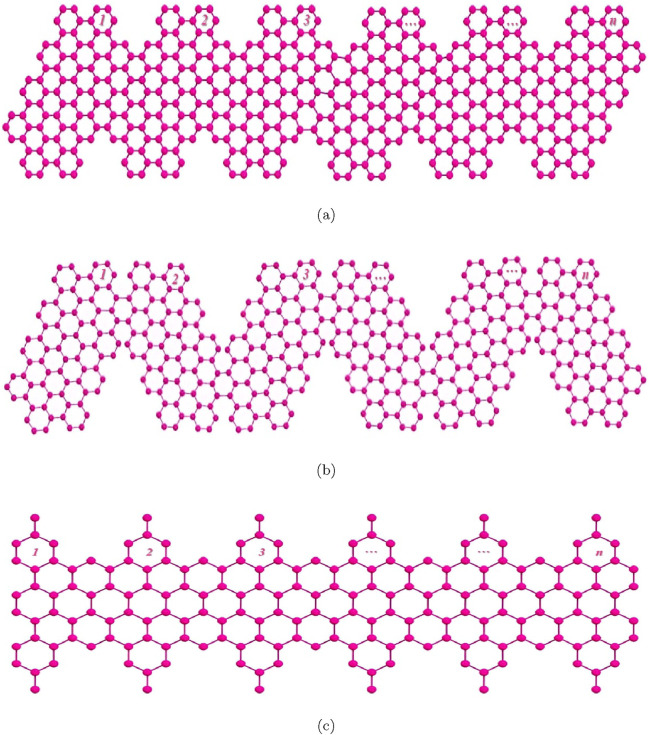
Molecular graph of Graphene nanoribbons growth $n^{th}$ stage **(a)** p-ANR; **(b)** Zig-zag p-ANR; **(c)** 4-CNR.

**FIGURE 2:**
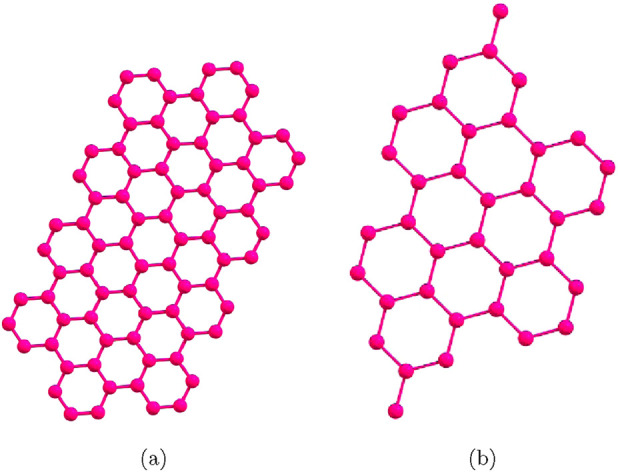
Molecular graph of Graphene nanoribbons unit cells **(a)** both p-ANR and Zig-zag p-ANR; **(b)** 4-CNR.

Compared with the conventional semiconductors silicon, and gallium arsenide, graphene is superior in electrical conductivity, thermal conductivity, and mechanical strength (Son et al., 2006; Sorokin and Chernozatonskii, 2013). Graphene nanoribbon (GNR) are single-layer of graphene with width smaller than 50 nm, and have distinctive electronic and optical characteristics resulting from the nanostructure and edge carbon atoms. It has been identified that, while bulk graphene produces no bandgap, GNRs can be electronically modulated to be semi-conductive with bandgap depending on the width and edge geometry. The structure of edge of a GNR is either zigzag or armchair, which also composes the characteristics of the GNR: zigzag edge exhibits magnetic property, while the width of the bandgap will be affected by the armchair edge. In this paper, we are mainly studied on the topological properties of three graphene nanoribbons structures, namely, p-ANR, Zig-zag p-ANR and 4-CNR through machine learning analysis on degree-based topological indices of these structures. Graphical structure of these three GNRs are portrayed in the Figure 1. The p-ANR exhibits a structure characterized by an N = 9 armchair GNR, featuring partially extended edges that reach a maximum width of N = 15 armchair GNR (Figure 1a), with N representing the number of carbon atoms across the nanoribbon. This geometry shows a near infrared gap. And for the Zig-zag p-ANR a zig-zag geometrical orientation of the p-ANR molecule is constituted (Figure 1b). However, the 4-CNR is derived from N = 4 zigzag edge GNRs that integrate additional benzo-fused rings. It takes the shape of a cove-type edge configuration (Figure 1c), which results in a visible gap-filled semiconducting nanostructure. The unit cells of these three graphene nanoribbons are given in Figure 2, for p-ANR and zog-zag p-ANR the unit cell are same Figures 2a,b represents the primary cell of 4-CNR structure (Bai and Huang, 2010; Jordan et al., 2016; Liu et al., 2015; Tiwari et al., 2020; Zhao et al., 2017).

A branch of mathematical chemistry known as chemical graph theory, which studies the advanced uses of graph theory in finding solutions of molecular phenomena. Here, a molecule is represented as a graph known as molecular graph. As a fundamental element in contemporary chemistry, chemical graph theory offers a systematic approach to depicting, evaluating, and forecasting the actions of chemical compounds. The primary aim of chemical graph theory is to utilize algebraic properties to reduce the topological structure of a molecule into a numerical value. One of these graph invariants are topological indices, they are numerical measures used in chemical graph theory to explain the structure of molecules based primarily on their connectivity. It was introduced by Harold Weiner in 1947 while he was working on the boiling point of alkanes. They offer valuable insights into the arrangement of atoms in molecules, which is essential for understanding their characteristics and behaviours. By examining how atoms are connected in a molecule, topological indices encode significant structural details that can be linked to various physical and chemical properties as well as biological properties. The stability, reactivity, and other physical characteristics of molecules can be better understood with the use of topological indices. Because of their many uses in advancing quantitative structure-activity relationship (QSAR) and quantitative structure-property relationship (QSPR) research, topological descriptors have been the subject of much research in recent years. They are widely employed in computational chemistry, various drug development, and chemical data analysis for diverse purposes. The elegant concepts of topological indices (TIs) or molecular descriptors can assist in the surmounting of the aforementioned limitations by further fine-tuning of the synthesis routes, improving the material stability, and controlling the bandgap. Using these mathematical descriptors, researchers are able build up better performance in production, understand strength and structural integrity and also be able to form specific functionalization strategies for graphene. According to models with developed topological indices, researchers can save time and money and quickly introduce graphene-based materials. This approach is important for the development of graphene for different applications since they provide information about structure-property relationship (Balaban, 1985; Gutman, 2006; Gutman, 2013; Trinajstic, 2018; Wiener, 1947).

Degree-based topological indices encode the coordination environment and connectivity distribution of atoms within a molecular graph, which are fundamental structural determinants of the electronic behaviour of graphene nanoribbons. In GNRs, edge configuration and vertex coordination strongly influence π -electron delocalization, bandgap opening, and transport characteristics. The indices considered in this study capture these features at different levels of structural resolution. Zagreb-type indices (Bi-Zagreb and Tri-Zagreb) quantify pairwise and higher-order interactions between vertex degrees, thereby reflecting the uniformity of carbon coordination along the nanoribbon backbone and edges. Higher Zagreb index values correspond to more regular coordination patterns, which are associated with enhanced π -electron delocalization and a higher likelihood of finite bandgap formation in bottom-up synthesized GNRs. The Randić and Sum Connectivity indices emphasize inverse degree relationships and connectivity efficiency, capturing how electron pathways are distributed across the nanoribbon network. These indices have been widely used as proxies for molecular branching and electronic communication efficiency, making them relevant for distinguishing metallic from semiconducting ribbon topologies. In contrast, harmonic and harmonic-geometric indices weight low-degree vertices more strongly, accentuating coordination heterogeneity and edge irregularity. In graphene nanoribbons, such heterogeneity can disrupt conjugation continuity and reduce electronic coherence, which is consistent with their observed negative contribution in the classification analysis. These numerical descriptors including Zagreb, Randić, and Atom bond connectivity indices are used to predict stability, conductivity, and other important parameters of semiconductor material which supports new material finding, understanding structure-property relationship and device performance enhancement. The first Zagreb index is one of the general topological indexes that are applicable in numerous branches of science and technology, and are most popular in the field of chemical, pharmacology, and material science. It is useful in predicting the boiling point of chemical compounds, especially benzenoid hydrocarbons, and is greatly involved in drug design in the processes of evaluating and predicting drug properties. It plays an important role in materials science where the researcher needs to explain the structure as well as behaviour of new as well as advanced materials. Further, it aids chemical characterization by associating chemical structures with chemical properties and behaviour (Augustine and Roy, 2022; Balaban and Ivanciuc, 2000; Fath-Tabar et al., 2010; Gayathri and Roy, 2025; Hayat et al., 2019; Hosoya and Hosoi, 1976; Jeyaraj and Roy, 2023; Jyothish et al., 2024; Jyothish and Roy, 2024).

Machine learning, as a data-driven extension of artificial intelligence, has increasingly been integrated with chemical graph theory to extract structure-property relationships from topological descriptors. In molecular and materials chemistry, established indices such as the Zagreb, Randić, Sombor, and related degree-based descriptors have been shown to encode connectivity patterns, coordination regularity, and structural compactness, which are directly relevant to electronic behaviour in conjugated systems. When combined with interpretable machine learning models, these descriptors enable systematic analysis of how graph topology influences material properties. In the context of graphene-based materials, machine learning offers a framework to examine how variations in nanoribbon structure and edge topology affect semiconducting behaviour, including trends associated with π -electron delocalization and connectivity efficiency. Among supervised learning approaches, classification methods are particularly suited for distinguishing electronic behaviour classes when direct prediction of continuous properties such as bandgap values is not the primary objective. Logistic regression, in particular, provides an interpretable model that links descriptor contributions directly to class discrimination. Model evaluation using Receiver Operating Characteristic (ROC) analysis further enables quantitative assessment of classification performance and robustness. In this work, machine learning is employed not as a generic predictive tool, but as an interpretable framework grounded in chemical graph theory. By defining a physically motivated Composite Semiconducting Index (CSI) derived from well-established degree-based descriptors, the study aims to connect topological features of bottom-up synthesized graphene nanoribbons with their semiconducting propensity, providing a structure-driven screening approach rather than speculative statistical correlation (Jordan and Mitchell, 2015; Karthikeyan and Priyakumar, 2022; Raihan, 2023; Sarker, 2021; Barman and Sarkar, 2025; Gayathri et al., 2025).

## 2 Materials and methods

In chemical graph theory, A molecular graph G=(V(G),E(G)) is a simple graph, where the edges indicate covalent bonds between the associated atoms and the nodes represent atoms. Specifically, hydrocarbons are made up of only hydrogen and carbon atoms, and the carbon skeleton of the molecule is represented by its molecular graph. Topological indices are numerical quantities that are derived from these molecular graphs, which delineate the structural features of the molecules, different degree-based topological indices and their mathematical equations are given in Tables 1, 2. The total count of edges that connect to the vertex p∈V(G) is termed the degree of vertex p , denoted as $d_{p}$ (Bondy and Murty, 1976; Trinajstic, 2018).

**TABLE 1:** Degree-based topological descriptors.

First zagreb	$M_{1}\left(G\right)$ = $\underset{pq\in E\left(G\right)}{\sum }\;\left[d_{p}+d_{q}\right]$
Second zagreb	$M_{2}\left(G\right)=\underset{pq\in E\left(G\right)}{\sum }\;\;\left[d_{p}\times d_{q}\right]$
Randić	$R\left(G\right)=\underset{pq\in E\left(G\right)}{\sum }\;\;\left[\frac{1}{\sqrt{d_{p}d_{p}}}\right]$
Harmonic	$H\left(G\right)=\underset{pq\in E\left(G\right)}{\sum }\;\;\left[\frac{2}{d_{p}+d_{q}}\right]$
Sum connectivity	$SC\left(G\right)=\underset{pq\in E\left(G\right)}{\sum }\;\;\frac{1}{\sqrt{d_{p}+d_{q}}}$
Geometric arithmetic	$GA\left(G\right)=\underset{pq\in E\left(G\right)}{\sum }\;\;\frac{2\sqrt{d_{p}d_{q}}}{d_{p}+d_{q}}$
Inverse sum	$IS\left(G\right)=\underset{pq\in E\left(G\right)}{\sum }\;\;\frac{d_{p}d_{q}}{d_{p}+d_{q}}$
Symmetric division	$SDI\left(G\right)=\underset{pq\in E\left(G\right)}{\sum }\;\;\frac{{d_{p}}^{2}+{d_{q}}^{2}}{d_{p}d_{q}}$
Atom bond connectivity	$ABC\left(G\right)=\underset{pq\in E\left(G\right)}{\sum }\;\;\sqrt{\frac{d_{p}+d_{q}-2}{d_{p}d_{q}}}$
Third zagreb	$M_{3}\left(G\right)=\underset{pq\in E\left(G\right)}{\sum }|d_{p}-d_{q}|$
Reduced second zagreb	$RM_{2}\left(G\right)=\underset{pq\in E\left(G\right)}{\sum }\left(d_{p}-1\right)\left(d_{q}-1\right)$
Hyper zagreb	$HM\left(G\right)=\underset{pq\in E\left(G\right)}{\sum }{\left(d_{p}+d_{q}\right)}^{2}$
Reciprocal randić	$RR\left(G\right)=\underset{pq\in E\left(G\right)}{\sum }\sqrt{d_{p}d_{q}}$
Reduced reciprocal randić	$RRR\left(G\right)=\underset{pq\in E\left(G\right)}{\sum }\sqrt{\left(d_{p}-1\right)\left(d_{q}-1\right)}$
Forgotten	$F\left(G\right)=\underset{pq\in E\left(G\right)}{\sum }d_{p}^{2}+d_{q}^{2}$

**TABLE 2:** Degree-based topological descriptors.

Bi-zagreb	$BM\left(G\right)=\underset{pq\epsilon E\left(G\right)}{\sum }\left(d_{p}+d_{q}+d_{p}d_{q}\right)$
Tri-zagreb	$TM\left(G\right)=\underset{pq\epsilon E\left(G\right)}{\sum }\left({d^{2}}_{p}+{d^{2}}_{q}+d_{p}d_{q}\right)$
Geometric-harmonic	$GH\left(G\right)=\underset{pq\epsilon E\left(G\right)}{\sum }\frac{\sqrt{d_{p}d_{q}}\left(d_{p}d_{q}\right)}{2}$
Geometric-bi zagreb	$GBM\left(G\right)=\underset{pq\epsilon E\left(G\right)}{\sum }\frac{\sqrt{d_{p}d_{q}}}{d_{p}+d_{q}+d_{p}d_{q}}$
Geometric-tri zagreb	$GTM\left(G\right)=\underset{pq\epsilon E\left(G\right)}{\sum }\frac{\sqrt{d_{p}d_{q}}}{{d^{2}}_{p}+{d^{2}}_{q}+d_{p}d_{q}}$
Harmonic-geometric	$HG\left(G\right)=\underset{pq\epsilon E\left(G\right)}{\sum }\frac{2}{\sqrt{d_{p}d_{q}}\left(d_{p}+d_{q}\right)}$
Harmonic-bi zagreb	$HBM\left(G\right)=\underset{pq\epsilon E\left(G\right)}{\sum }\frac{2}{\left(d_{p}d_{q}\right)\left(d_{p}+d_{q}+d_{p}d_{q}\right)}$
Harmonic-tri zagreb	$HTM\left(G\right)=\underset{pq\epsilon E\left(G\right)}{\sum }\frac{2}{\left(d_{p}d_{q}\right)\left({d^{2}}_{p}+{d^{2}}_{q}+d_{p}d_{q}\right)}$
Bi zagreb-geometric	$BMG\left(G\right)=\underset{pq\epsilon E\left(G\right)}{\sum }\frac{d_{p}+d_{q}+d_{p}d_{q}}{\sqrt{d_{p}d_{q}}}$
Bi zagreb-harmonic	$BMH\left(G\right)=\underset{pq\epsilon E\left(G\right)}{\sum }\frac{\left(d_{p}d_{q}\right)\left(d_{p}+d_{q}+d_{p}d_{q}\right)}{2}$
Tri zagreb-geometric	$TMG\left(G\right)=\underset{pq\epsilon E\left(G\right)}{\sum }\frac{{d^{2}}_{p}+{d^{2}}_{q}+d_{p}d_{q}}{\sqrt{d_{p}d_{q}}}$
Tri zagreb-harmonic	$TMH\left(G\right)=\underset{pq\epsilon E\left(G\right)}{\sum }\frac{\left(d_{p}d_{q}\right)\left({d^{2}}_{p}+{d^{2}}_{q}+d_{p}d_{q}\right)}{2}$
Sombor	$SO\left(G\right)=\underset{pq\in E\left(G\right)}{\sum }\sqrt{d_{p}^{2}+d_{q}^{2}}$
Augmented zagreb	$AZ\left(G\right)=\underset{pq\in E\left(G\right)}{\sum }{\left[\frac{d_{p}d_{q}}{d_{p}+d_{q}-2}\right]}^{3}$
Modified second zagreb	$M_{2}^{*}\left(G\right)=\underset{pq\in E\left(G\right)}{\sum }\frac{1}{d_{p}d_{q}}$

For ensuring a chemically interpretable machine learning framework, a Composite Semiconducting Index (CSI) was defined and employed as the target variable. The CSI is constructed as the arithmetic mean of four established degree-based topological descriptors that have been independently reported to correlate with electronic delocalization and structural regularity in conjugated carbon systems:

$$CSI=\frac{1}{4}\left(SO+GA+R+SC\right)$$ (1)

These indices collectively capture coordination uniformity, bond connectivity efficiency, and topological compactness, which are structural characteristics closely associated with π -electron delocalization and semiconducting behaviour in graphene-based nanostructures (Gutman, 2021; Randić, 1975; Vukičević and Furtula, 2009; Zhou and Trinajstić, 2009). Normalization was applied prior to aggregation to ensure equal contribution from each descriptor. Logistic regression was employed to model the relationship between degree-based topological descriptors and the CSI (Equation 1) Predicted probabilities for semiconducting behaviour were obtained from the trained logistic regression model using the CSI-based classification framework. For each graphene nanoribbon structure, the model outputs a posterior probability P (semiconducting), reflecting the likelihood that a given topology belongs to the semiconducting class. To visualize class separability and model confidence, probability distributions were analysed separately for semiconducting and metallic nanoribbons. Histograms were constructed using normalized frequencies to allow direct comparison between classes. In addition, kernel density estimation (KDE) was applied to generate smooth probability density curves, providing a continuous representation of classification confidence across the probability space and a decision threshold of 0.5 was used for binary classification. Model performance was evaluated using Receiver Operating Characteristic (ROC) analysis, with the area under the curve (AUC) used as the primary performance metric.

For the computation of topological indices, we have employed edge partition approach and graph theoretical techniques. Using Matlab software, we generated the topological indices general formula. The structural configurations of the graphene nanoribbon structures were plotted using Chemcraft, and the numerical findings were visually represented using Origin software.

## 3 Main results

This section summarizes the main findings of the study. We have calculated the generalized mathematical expressions for the topological indices based on the degrees of the vertices for the graphene nano-ribbons p-ANR, zigzag p-ANR, and 4-CNR. And the numerical analysis of these computed topological indices has done using the Matlab software.

### 3.1 Results on p-ANR

Let $G_{1}$ be the corresponding molecular graph of the p-ANR structure, which have vertices of degrees 2 and 3. So there will be edges with end vertex degrees (2,2), (2,3) and (3,3), and the corresponding edge partition based on these degree is given in Table 3. The total number of vertices and edges of p-ANR are |V|=78n and |E|=109n−5 respectively. Also, the vertex count for degree 2 and 3 of the chemical graph of the p-ANR structure are $|V_{2}|=16n+10$ and $|V_{3}|=62n-10$ respectively.

**TABLE 3:** Edge partition of p-ANR.

$E_{\left(d_{r},d_{s}\right)}$	$\left|E_{\left(d_{r},d_{s}\right)}\right|$
$E_{\left(2,2\right)}$	8n+8
$E_{\left(2,3\right)}$	16n+4
$E_{\left(3,3\right)}$	85n−17

Theorem 3.1 Let $G_{1}$ represents the graph of p-ANR(n); n≥1 structure. Then,

-
$BM\left(G_{1}\right)=1515n-147$

-
$TM\left(G_{1}\right)=2695n-287$

-
$GH\left(G_{1}\right)=797n+\frac{5\sqrt{6}\left(16n+4\right)}{2}-121$

-
$GBM\left(G_{1}\right)=19n+\frac{\sqrt{6}\left(16n+4\right)}{11}-\frac{7}{5}$

-
$GTM\left(G_{1}\right)=\frac{97n}{9}+\frac{\sqrt{6}\left(16n+4\right)}{19}-\frac{5}{9}$

-
$HG\left(G_{1}\right)=\frac{103n}{9}+\frac{\sqrt{2}\sqrt{3}\left(16n+4\right)}{15}+\frac{1}{9}$

-
$HBM\left(G_{1}\right)=\frac{2941n}{990}+\frac{53}{198}$

-
$HTM\left(G_{1}\right)=\frac{13232n}{7695}+\frac{1598}{7695}$

-
$BMG\left(G_{1}\right)=457n+\frac{11\sqrt{6}\left(16n+4\right)}{6}-53$

-
$BMH\left(G_{1}\right)=4393n-527$

-
$TMG\left(G_{1}\right)=813n+\frac{19\sqrt{6}\left(16n+4\right)}{6}-105$

-
$TMH\left(G_{1}\right)=7837n-995$

-
$M_{1}\left(G_{1}\right)=622n-50$

-
$M_{2}\left(G_{1}\right)=893n-97$

-
$R\left(G_{1}\right)=\frac{97n}{3}+\frac{\sqrt{6}\left(16n+4\right)}{6}-\frac{5}{3}$

-
$H\left(G_{1}\right)=\frac{581n}{15}-\frac{1}{15}$

-
$SC\left(G_{1}\right)=4n+\frac{5\sqrt{2}\left(16n+4\right)}{5}+\frac{5\sqrt{3}\left(85n-17\right)}{6}+4$

-
$GA\left(G_{1}\right)=93n+\frac{2\sqrt{6}\left(16n+4\right)}{5}-9$

-
$IS\left(G_{1}\right)=\frac{1547n}{10}-\frac{127}{10}$

-
$AZI\left(G_{1}\right)=\frac{662n}{3}-\frac{28}{3}$

-
$ABC\left(G_{1}\right)=\frac{170n}{3}+\frac{2\sqrt{2}\left(8n+8\right)}{2}+\frac{2\sqrt{2}\left(16n+4\right)}{2}-\frac{34}{3}$

-
$HM\left(G_{1}\right)=3588n-384$

-
$M_{3}\left(G_{1}\right)=16n+4$

-
$RM_{2}\left(G_{1}\right)=380n-52$

-
$RR\left(G_{1}\right)=271n+\sqrt{6}\left(16n+4\right)-35$

-
$RRR\left(G_{1}\right)=178n+\sqrt{2}\left(16n+4\right)-26$

-
$F\left(G_{1}\right)=1802n-190$

-
$M_{2}^{*}\left(G_{1}\right)=\frac{127n}{9}+\frac{7}{9}$

-
$SO\left(G_{1}\right)=2\sqrt{2}\left(8n+8\right)+\sqrt{13}\left(16n+4\right)+3\sqrt{2}\left(85n-17\right)$

-
$AZ\left(G_{1}\right)=\frac{74253n}{64}-\frac{6249}{64}$

### 3.2 Results on zigzag p-ANR

For the structure zigzag p-ANR, let $G_{2}$ be the corresponding molecular graph with vertices having degrees 2 and 3, and hence the partition of edge set contains edges with classes (2,2), (2,3) and (3,3), which is given in Table 4. The total number of vertices and edges of zigzag p-ANR are |V|=72n+6 and |E|=98n+6 respectively. And also the vertex count with degree 2 and 3 are $|V_{2}|=20n+6$ and $|V_{3}|=52n$ respectively.

**TABLE 4:** Edge partition of zigzag p-ANR.

$E_{\left(d_{r},d_{s}\right)}$	$\left|E_{\left(d_{r},d_{s}\right)}\right|$
$E_{\left(2,2\right)}$	12n+4
$E_{\left(2,3\right)}$	16n+4
$E_{\left(3,3\right)}$	70n−2

Theorem 3.2 Let $G_{2}$ represents the graph of zigzag p-ANR(n); n≥1 structure. Then,

-
$BM\left(G_{2}\right)=1322n+46$

-
$TM\left(G_{2}\right)=2338n+70$

-
$GH\left(G_{2}\right)=678n+\frac{5\sqrt{2}\sqrt{3}\left(16n+4\right)}{2}-2$

-
$GBM\left(G_{2}\right)=17n+\frac{\sqrt{6}\left(16n+4\right)}{11}+\frac{3}{5}$

-
$GTM\left(G_{2}\right)=\frac{88n}{9}+\frac{\sqrt{6}\left(16n+4\right)}{19}+\frac{4}{9}$

-
$HG\left(G_{2}\right)=\frac{97n}{9}+\frac{\sqrt{2}\sqrt{3}\left(16n+4\right)}{15}+\frac{7}{9}$

-
$HBM\left(G_{2}\right)=\frac{5717n}{1980}+\frac{139}{396}$

-
$HTM\left(G_{2}\right)=\frac{26179n}{15390}+\frac{3481}{15390}$

-
$BMG\left(G_{2}\right)=398n+\frac{11\sqrt{6}\left(16n+4\right)}{6}+6$

-
$BMH\left(G_{2}\right)=3782n+84$

-
$TMG\left(G_{2}\right)=702n+\frac{19\sqrt{6}\left(16n+4\right)}{6}+6$

-
$TMH\left(G_{2}\right)=6718n+124$

-
$M_{1}\left(G_{2}\right)=548n+24$

-
$M_{2}\left(G_{2}\right)=774n+22$

-
$R\left(G_{2}\right)=\frac{88n}{3}+\frac{\sqrt{6}\left(16n+4\right)}{6}+\frac{4}{3}$

-
$H\left(G_{2}\right)=\frac{536n}{15}+\frac{44}{15}$

-
$SC\left(G_{2}\right)=6n+\frac{5\sqrt{2}\left(16n+4\right)}{5}+\frac{5\sqrt{3}\left(70n-2\right)}{6}+2$

-
$GA\left(G_{2}\right)=82n+\frac{2\sqrt{6}\left(16n+4\right)}{5}+2$

-
$IS\left(G_{2}\right)=\frac{681n}{5}+\frac{29}{5}$

-
$AZI\left(G_{2}\right)=\frac{596n}{3}+\frac{38}{3}$

-
$ABC\left(G_{2}\right)=\frac{140n}{3}+\frac{2\sqrt{2}\left(12n+4\right)}{2}+\frac{2\sqrt{2}\left(16n+4\right)}{2}-\frac{4}{3}$

-
$HM\left(G_{2}\right)=3112n+92$

-
$M_{3}\left(G_{2}\right)=16n+4$

-
$RM_{2}\left(G_{2}\right)=324n+4$

-
$RR\left(G_{2}\right)=234n+\sqrt{6}\left(16n+4\right)+2$

-
$RRR\left(G_{2}\right)=152n+\sqrt{2}\left(16n+4\right)$

-
$F\left(G_{2}\right)=1564n+48$

-
$M_{2}^{*}\left(G_{2}\right)=\frac{121n}{9}+\frac{13}{9}$

-
$SO\left(G_{2}\right)=2\sqrt{2}\left(12n+4\right)+\sqrt{13}\left(16n+4\right)+3\sqrt{2}\left(70n-2\right)$

-
$AZ\left(G_{2}\right)=\frac{32683n}{32}-\frac{1319}{32}$

### 3.3 Results on 4-CNR

In the case of 4-CNR, $G_{3}$ be the molecular graph of the 4-CNR structure with vertices having degrees 1,2 and 3, and hence edge partition contains edges in classes (1,3), (2,2), (2,3) and (3,3), which is given in Table 5. The total number of vertices and edges of zigzag p-ANR are |V|=32n+24 and |E|=43n+28 respectively. And also the vertex count with degree 1,2 and 3 are $|V_{1}|=2n+2$ , $|V_{2}|=6n+12$ , and $|V_{3}|=24n+10$ respectively.

**TABLE 5:** Edge partition of 4-CNR.

$E_{\left(d_{r},d_{s}\right)}$	$\left|E_{\left(d_{r},d_{s}\right)}\right|$
$E_{\left(1,3\right)}$	2n+2
$E_{\left(2,2\right)}$	5
$E_{\left(2,3\right)}$	12n+14
$E_{\left(3,3\right)}$	29n+7

Theorem 3.3 Let $G_{3}$ represents the graph of 4-CNR; n≥1 structure. Then,

-
$BM\left(G_{3}\right)=581n+313$

-
$TM\left(G_{3}\right)=1037n+541$

-
$GH\left(G_{3}\right)=261n+\frac{3\sqrt{3}\left(2n+2\right)}{2}+\frac{5\sqrt{2}\sqrt{3}\left(12n+14\right)}{2}+83$

-
$GBM\left(G_{3}\right)=\frac{29n}{5}+\frac{\sqrt{3}\left(2n+2\right)}{7}+\frac{\sqrt{6}\left(12n+14\right)}{11}+\frac{53}{20}$

-
$GTM\left(G_{3}\right)=\frac{29n}{9}+\frac{\sqrt{3}\left(2n+2\right)}{13}+\frac{\sqrt{6}\left(12n+14\right)}{19}+\frac{29}{18}$

-
$HG\left(G_{3}\right)=\frac{29n}{9}+\frac{\sqrt{3}\left(2n+2\right)}{6}+\frac{\sqrt{2}\sqrt{3}\left(12n+14\right)}{15}+\frac{73}{36}$

-
$HBM\left(G_{3}\right)=\frac{881n}{693}+\frac{64733}{55440}$

-
$HTM\left(G_{3}\right)=\frac{71347n}{100035}+\frac{553837}{800280}$

-
$BMG\left(G_{3}\right)=145n+\frac{7\sqrt{3}\left(2n+2\right)}{3}+\frac{11\sqrt{6}\left(12n+14\right)}{6}+55$

-
$BMH\left(G_{3}\right)=1656n+801$

-
$TMG\left(G_{3}\right)=261n+\frac{13\sqrt{3}\left(2n+2\right)}{3}+\frac{19\sqrt{6}\left(12n+14\right)}{6}+93$

-
$TMH\left(G_{3}\right)=2958n+1391$

-
$M_{1}\left(G_{3}\right)=242n+140$

-
$M_{2}\left(G_{3}\right)=339n+173$

-
$R\left(G_{3}\right)=\frac{29n}{3}+\frac{\sqrt{3}\left(2n+2\right)}{3}+\frac{\sqrt{6}\left(12n+14\right)}{6}+\frac{29}{6}$

-
$H\left(G_{3}\right)=\frac{232n}{15}+\frac{343}{30}$

-
$SC\left(G_{3}\right)=n+\frac{\sqrt{5}\left(12n+14\right)}{5}+\frac{\sqrt{6}\left(29n+7\right)}{6}+\frac{7}{2}$

-
$GA\left(G_{3}\right)=29n+\frac{\sqrt{3}\left(2n+2\right)}{2}+\frac{2\sqrt{6}\left(12n+14\right)}{5}+12$

-
$IS\left(G_{3}\right)=\frac{297n}{5}+\frac{169}{5}$

-
$AZI\left(G_{3}\right)=\frac{272n}{3}+61$

-
$ABC\left(G_{3}\right)=\frac{58n}{3}+\frac{5\sqrt{2}}{2}+\frac{\sqrt{2}\left(12n+14\right)}{2}+\frac{\sqrt{2}\sqrt{3}\left(2n+2\right)}{3}+\frac{14}{3}$

-
$HM\left(G_{3}\right)=1376n+714$

-
$M_{3}\left(G_{3}\right)=16n+18$

-
$RM_{2}\left(G_{3}\right)=140n+61$

-
$RR\left(G_{3}\right)=87n+\sqrt{3}\left(2n+2\right)+\sqrt{6}\left(12n+14\right)+31$

-
$RRR\left(G_{3}\right)=58n+\sqrt{2}\left(12n+14\right)+19$

-
$F\left(G_{3}\right)=698n+368$

-
$M_{2}^{*}\left(G_{3}\right)=\frac{53n}{9}+\frac{181}{36}$

-
$SO\left(G_{3}\right)=10\sqrt{2}+\sqrt{10}\left(2n+2\right)+3\sqrt{2}\left(29n+7\right)+\sqrt{13}\left(12n+14\right)$

-
$AZ\left(G_{3}\right)=\frac{27717n}{64}-\frac{15263}{64}$

### 3.4 Numerical computation

**FIGURE 3:**
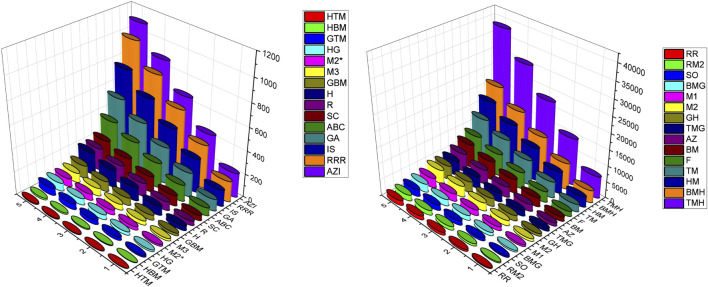
Visual representation of topological descriptors values of p-ANR.

**FIGURE 4:**
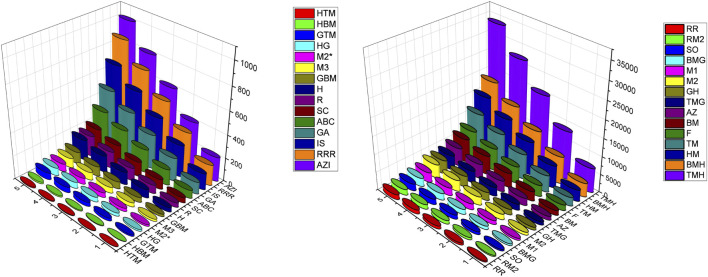
Visual representation of topological descriptors values of zig-zag p-ANR.

**FIGURE 5:**
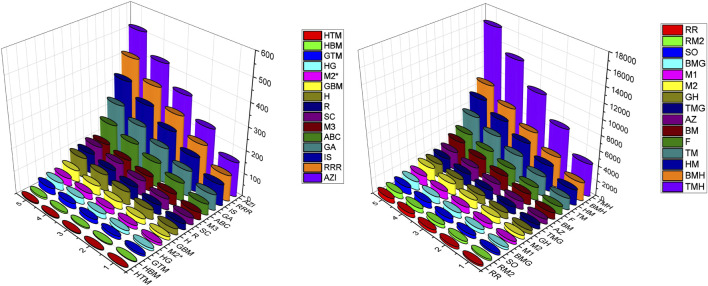
Visual representation of topological descriptors values of 4-CNR.

In this section we have computed the numerical values of the various degree based topological of the structures p-ANR, zigzag p-ANR and 4-CNR for the dimensions n=1 to 5. From analyzing these Tables 6–8, we can see that values of the topological indices are increasing with the dimension of the graphene nanoribbons. Also out of these computed thirty indices the TMH -index shows the highest numerical values for the considered three graphene nanoribbons of each dimension and HTM -index has the lowest numerical values also. The graphical representation of these topological indices values in Tables 6–8 are given in the Figures 3–5 respectively, we can see these observations in those descriptors 3D plots.

**TABLE 6:** Quantitative values for degree-based topological indices of p-ANR.

Indices	$M_{1}$	$M_{2}$	R	H	SC	GA	IS	AZI	ABC	HM	$M_{3}$	$RM_{2}$	RR	RRR	F
n = 1	572	796	38.8316	38.6667	44.7052	103.5959	142	211.3333	70.7892	3204	20	328	284.9898	180.2843	1612
n = 2	1194	1689	77.6969	77.4000	90.5617	212.2727	297	432.000	144.4264	6792	36	708	595.1816	380.9117	3414
n = 3	1816	2582	116.5622	116.1333	136.4182	320.9494	451	652.6667	218.0636	10380	52	1088	905.3735	581.5391	5216
n = 4	2438	3475	155.4276	154.8667	182.2747	429.6261	606	873.3333	291.7009	13968	68	1468	1215.5653	782.1665	7018
n = 5	3060	4368	194.2929	193.6000	228.1312	538.3029	761	1094.000	365.3381	17556	84	1848	1525.7571	982.7939	8820

Indices	BM	TM	GH	GBM	GTM	HG	HBM	HTM	BMG	BMH	TMG	TMH	SO	AZ	$M_{2}^{*}$
n = 1	1368	2408	798.4745	22.0536	12.8006	14.8215	3.2384	1.9272	493.8146	3866	863.1344	6842	405.8654	1062.5625	14.8889
n = 2	2883	5103	1693.4541	44.6165	25.6411	28.8788	6.2091	3.6468	1022.6663	8259	1800.2418	14679	846.8061	2222.7656	29.0000
n = 3	4398	7798	2588.4337	67.1794	38.4816	42.9360	9.1798	5.3663	1551.5180	12652	2737.3493	22516	1287.7468	3382.9688	43.1111
n = 4	5913	10493	3483.4133	89.7423	51.3222	56.9932	12.1505	7.0859	2080.3697	17045	3674.4568	30353	1728.6875	4543.1719	57.2222
n = 5	7428	13188	4378.3928	112.3052	64.1627	71.0505	15.1212	8.8055	2609.2214	21438	4611.5643	38190	2169.6282	5703.3750	71.3333

**TABLE 7:** Quantitative values for degree-based topological indices of zigzag p-ANR.

Indices	$M_{1}$	$M_{2}$	R	H	SC	GA	IS	AZI	ABC	HM	$M_{3}$	$RM_{2}$	RR	RRR	F
n = 1	572	796	38.8316	38.6667	44.7052	103.5959	142	211.3333	70.7892	3204	20	328	284.9898	180.2843	1612
n = 2	1120	1570	74.6969	74.4000	86.4380	201.2727	278	410.0000	137.2548	6316	36	652	558.1816	354.9117	3176
n = 3	1668	2344	110.5622	110.1333	128.1708	298.9494	414	608.6667	203.7205	9428	52	976	831.3735	529.5391	4740
n = 4	2216	3118	146.4276	145.8667	169.9035	396.6261	551	807.3333	270.1861	12540	68	1300	1104.5653	704.1665	6304
n = 5	2764	3892	182.2929	181.6000	211.6363	494.3029	687	1006.0000	336.6518	15652	84	1624	1377.7571	878.7939	7868

Indices	BM	TM	GH	GBM	GTM	HG	HBM	HTM	BMG	BMH	TMG	TMH	SO	AZ	$M_{2}^{*}$
n = 1	1368	2408	798.4745	22.0536	12.8006	14.8215	3.2384	1.9272	493.8146	3866	863.1344	6842	405.8654	1062.5625	14.8889
n = 2	2690	4746	1574.4541	42.6165	24.6411	28.2121	6.1258	3.6283	963.6663	7648	1689.2418	13560	794.4802	2083.9063	28.3333
n = 3	4012	7084	2350.4337	63.1794	36.4816	41.6027	9.0131	5.3293	1433.5180	11430	2515.3493	20278	1183.0950	3105.2500	41.7778
n = 4	5334	9422	3126.4133	83.7423	48.3222	54.9932	11.9005	7.0303	1903.3697	15212	3341.4568	26996	1571.7098	4126.5938	55.2222
n = 5	6656	11760	3902.3928	104.3052	60.1627	68.3838	14.7879	8.7314	2373.2214	18994	4167.5643	33714	1960.3246	5147.9375	68.6667

**TABLE 8:** Quantitative values for degree-based topological indices of 4-CNR.

Indices	$M_{1}$	$M_{2}$	R	H	SC	GA	IS	AZI	ABC	HM	$M_{3}$	$RM_{2}$	RR	RRR	F
n = 1	382	512	27.4239	26.9000	30.8245	69.9388	93	151.6667	49.1863	2090	34	201	188.6149	113.7696	1066
n = 2	624	851	43.1442	42.3667	49.0303	112.4284	153	242.3333	78.6379	3466	50	341	308.4729	188.7401	1764
n = 3	866	1190	58.8646	57.8333	67.2360	154.9180	212	333.0000	108.0895	4842	66	481	428.3309	263.7107	2462
n = 4	1108	1529	74.5849	73.3000	85.4418	197.4076	271	423.6667	137.5411	6218	82	621	548.1889	338.6812	3160
n = 5	1350	1868	90.3052	88.7667	103.6475	239.8972	331	514.3333	166.9927	7594	98	761	668.0469	413.6518	3858

Indices	BM	TM	GH	GBM	GTM	HG	HBM	HTM	BMG	BMH	TMG	TMH	SO	AZ	$M_{2}^{*}$
n = 1	894	1578	513.6091	15.2294	8.7182	10.6505	2.4389	1.4053	332.9248	2457	585.6969	4349	273.2706	671.5625	10.9167
n = 2	1475	2615	853.2900	24.1965	13.7539	16.4096	3.7102	2.1185	539.8965	4113	954.7886	7307	445.8984	1104.6406	16.8056
n = 3	2056	3652	1192.9708	33.1635	18.7897	22.1688	4.9815	2.8317	746.8682	5769	1323.8803	10265	618.5261	1537.7188	22.6944
n = 4	2637	4689	1532.6517	42.1306	23.8254	27.9280	6.2528	3.5449	953.8399	7425	1692.9720	13223	791.1539	1970.7969	28.5833
n = 5	3218	5726	1872.3325	51.0976	28.8612	33.6871	7.5240	4.2582	1160.8115	9081	2062.0637	16181	963.7816	2403.8750	34.4722

## 4 Discussions

### 4.1 Logistic regression analysis using machine learning

**FIGURE 6:**
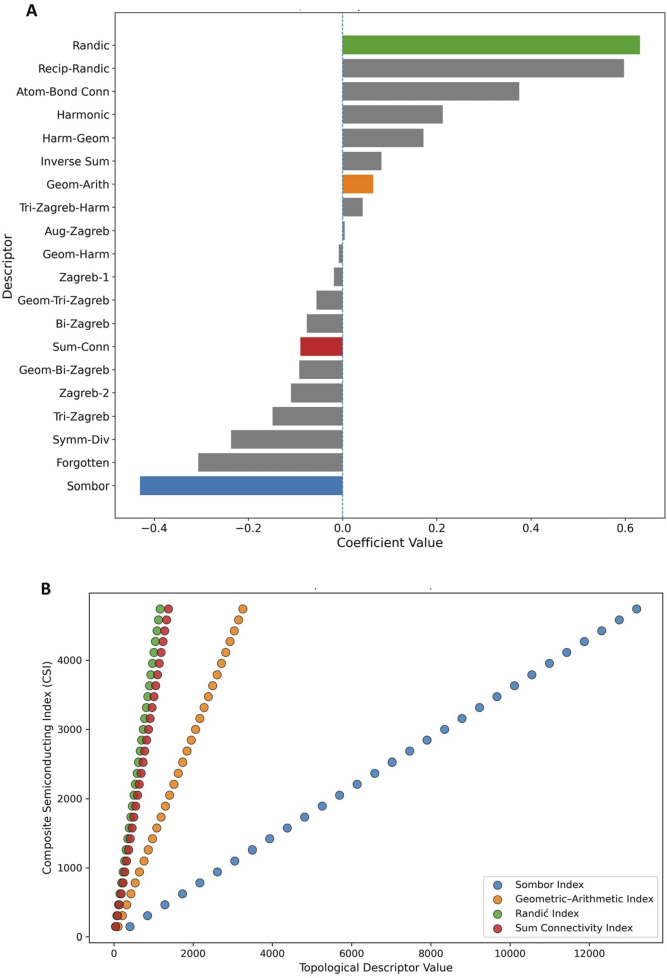
**(A)** Standardized logistic regression coefficients of topological descriptors used to classify semiconducting versus metallic graphene nanoribbons. Descriptors forming the Composite Semiconducting Index (CSI)-Sombor (blue), Geometric-Arithmetic (orange), Randić (green), and Sum Connectivity (red) are highlighted. **(B)** Correlation between individual CSI components and the Composite Semiconducting Index, illustrating their structured and monotonic contributions across nanoribbon sizes.

The logistic regression analysis reveals the relative influence of various graph-based indices such as the Bi-Zagreb, Tri-Zagreb, and harmonic-geometric indices on the predicted outcome. These indices capture mathematical properties that describe vertex degrees, geometric relationships, and harmonic interactions within the graph structure of the data, offering insights into the underlying structural features that may affect the target outcome (See Figure 6).

The logistic regression analysis (Figure 6A) identifies a physically interpretable subset of degree-based descriptors as dominant contributors to semiconducting classification. Among these, the Randić index exhibits the strongest positive coefficient, indicating that balanced vertex connectivity and coordination homogeneity strongly favour semiconducting behaviour in graphene nanoribbons. This observation is consistent with the role of Randić type descriptors in capturing efficient π -electron delocalization pathways along conjugated carbon frameworks. The Geometric-Arithmetic index also contributes positively, suggesting that structural regularity and bonding efficiency enhance the likelihood of finite bandgap formation. In contrast, the Sombor index shows a pronounced negative coefficient, implying that increased local degree variance and compact coordination environments correlate with metallic or near-zero bandgap characteristics. A similar, though weaker, negative contribution is observed for the Sum Connectivity index, indicating that excessive cumulative coordination may suppress semiconducting behaviour. These trends demonstrate that not all connectivity-enhancing descriptors act uniformly and that the nature of degree distribution is critical. Importantly, Figure 6B illustrates that the four highlighted descriptors contribute monotonically and systematically to the Composite Semiconducting Index (CSI), confirming that CSI reflects coordinated topological evolution rather than arbitrary statistical weighting. Collectively, these results show that simpler, degree-based indices capturing coordination balance and delocalization efficiency are more predictive of semiconducting behaviour than higher-order composite descriptors. This supports the use of interpretable, structure-derived indices for topology-driven screening of graphene nanoribbons with semiconducting potential.

### 4.2 Probability distribution analysis of semiconducting behaviour of GNRs

**FIGURE 7:**
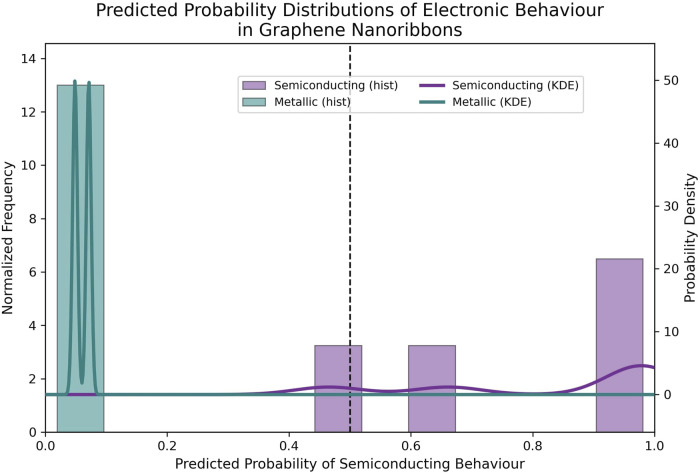
Distribution of predicted probabilities for semiconducting behaviour of graphene nanoribbons obtained from the CSI-based logistic regression model. Histograms and kernel density estimates compare semiconducting and metallic classes, with the vertical dashed line indicating the classification threshold.

Probability distribution analysis provides an interpretable means to evaluate model confidence and class separability beyond conventional accuracy metrics, which is particularly important when relating graph-theoretic descriptors to physically meaningful electronic behaviour. In this study, probability distributions are used to assess how effectively the CSI-based logistic regression model distinguishes semiconducting from metallic graphene nanoribbon topologies. Figure 7 shows a clear separation between the predicted probability distributions of semiconducting and metallic nanoribbons. Semiconducting structures are predominantly associated with high predicted probabilities, while metallic systems are confined to the low-probability region, with minimal overlap. This separation indicates that the classification is driven by structurally meaningful information encoded in the CSI rather than arbitrary statistical effects.

The smooth density profiles suggest a gradual transition between metallic and semiconducting behaviour, consistent with the continuous modulation of electronic properties in graphene nanoribbons as a function of edge topology and coordination patterns. The decision threshold lies within a low-density region, reducing ambiguity and supporting robust classification. Therefore, the systematic shift of the semiconducting distribution toward higher probabilities confirms the effectiveness of CSI as a cumulative descriptor integrating coordination balance and connectivity.

**FIGURE 8:**
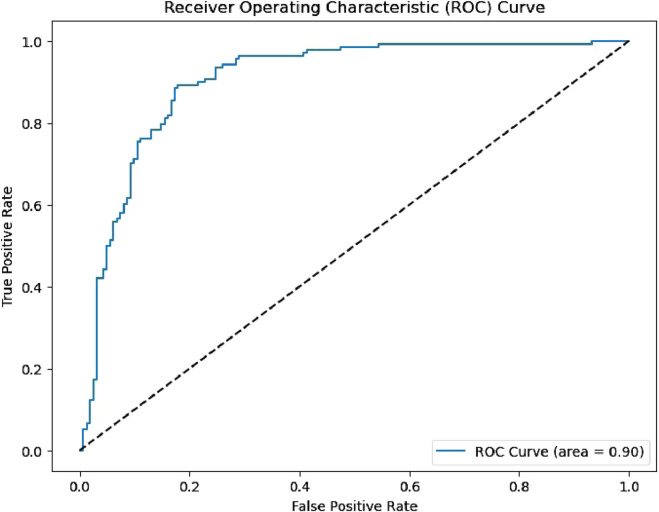
ROC curve.

These ROC curve (Figure 8) have practical implications for fields that use graph-based indices in predictive modeling, such as molecular graph analysis, network science, and social network analysis. For example, in chemical graph theory, the positive association of simpler Zagreb indices may suggest that molecular structures with higher vertex interactions are advantageous for semiconducting properties. Conversely, the negative influence of harmonic-geometric indices could indicate that specific structural characteristics detract from achieving the target outcome, which could guide future experimental designs to control for or avoid these features when possible. When considered alongside ROC performance, these results demonstrate that degree-based graph descriptors can reliably support topology-guided screening of GNRs.

### 4.3 Applications on topological study using machine learning methods

While logistic regression enables transparent interpretation of descriptor contributions, certain limitations remain. Multicollinearity among degree-based indices may influence coefficient magnitudes, as multiple descriptors encode overlapping structural information. Nevertheless, the probabilistic separation observed in the ROC and probability distribution analyses confirms the robustness of the composite CSI framework. From an application perspective, the present approach offers a topology-guided screening strategy rather than direct prediction of device-level properties. In the context of graphene nanoribbons, degree-based indices can serve as computationally inexpensive proxies for identifying structural motifs associated with semiconducting behaviour prior to detailed electronic structure calculations. Such pre-screening can support the rational selection of nanoribbon architectures for further theoretical or experimental evaluation, contributing to efficient design workflows in nanoscale semiconductor research. The logistic regression provides a valuable basis for interpreting feature importance; there are limitations that warrant further exploration. Multicollinearity among these indices may affect interpretability, as overlapping structural characteristics could skew the coefficient values. Future research could explore the robustness of these indices across different datasets, or expand the model by including interaction terms to deepen understanding of their roles.

Additionally, investigating whether these findings generalize to other domains or graph structures, as well as integrating these indices with advanced machine learning techniques such as ensemble methods or neural networks, may enhance predictive power and yield more comprehensive insights into the structural characteristics of the graph. This analysis of graph-based indices and their predictive significance can extend beyond molecular graph analysis and network science to applications in semiconductor device research. In semiconductor devices, such as transistors and integrated circuits, the structural and interaction properties of components at the nanoscale significantly impact performance. Graph based indices could be applied to model these interactions, capturing properties like electron mobility, conductivity pathways, and component interconnections in a semiconductor lattice.

## 5 Conclusion

In this work, to establish a physically motivated prediction task, a Composite Semiconducting Index (CSI) was introduced and employed within a logistic regression model. The resulting coefficient, probability distribution, and ROC analyses demonstrate that simpler degree-based descriptors, particularly Zagreb-type indices, contribute positively to the classification of semiconducting nanoribbon topologies. In contrast, harmonic-geometric descriptors exhibit weaker or negative influence, suggesting that excessive heterogeneity in local connectivity may reduce semiconducting likelihood. Overall, the results confirm that degree-based graph descriptors, when combined into a composite and interpretable target, can capture meaningful structural distinctions relevant to graphene nanoribbon electronic behaviour. This framework provides a computationally efficient, topology-guided screening strategy that can support structure-property analysis and guide further theoretical or experimental investigations in graphene-based nanomaterials.

## Data Availability

The original contributions presented in the study are included in the article/supplementary material, further inquiries can be directed to the corresponding author.

## References

[B1] Augustine T. Roy S. (2022). Topological study on triazine-based covalent-organic frameworks. Symmetry 14 (8), 1590. 10.3390/sym14081590

[B2] Bai J. Huang Y. (2010). Fabrication and electrical properties of graphene nanoribbons. Mater. Sci. Eng. R Rep. 70 (3-6), 341–353. 10.1016/j.mser.2010.06.019

[B3] Balaban A. T. (1985). Applications of graph theory in chemistry. J. Chemical Information Computer Sciences 25 (3), 334–343. 10.1021/ci00047a033

[B4] Balaban A. T. Ivanciuc O. (2000). “Historical development of topological indices,” in Topological indices and related descriptors in QSAR and QSPR (Taylor & Francis), 31–68.

[B5] Barman S. Sarkar U. (2025). Screening of potential candidates for solid electrolyte interphase materials for lithium-ion batteries through a data-driven approach. Phys. Chem. Chem. Phys. 27 (40), 21719–21738. 10.1039/d5cp02726h 41031462

[B6] Bondy J. A. Murty U. S. R. (1976). Graph theory with applications, 290. London: Macmillan. 10.1007/978-1-349-03521-2

[B7] Chae S. H. Lee Y. H. (2014). Carbon nanotubes and graphene towards soft electronics. Nano Converg. 1–26, 15. 10.1186/s40580-014-0015-5 28936384 PMC5591626

[B8] Fath-Tabar G. Furtula B. Gutman I. (2010). A new geometric-arithmetic index. J. Mathematical Chemistry 47, 477–486. 10.1007/s10910-009-9584-7

[B9] Gayathri K. B. Roy S. (2025). Quantitative structure property relationship study of postpartum depression medications using topological indices and regression models. Ain Shams Eng. J. 16 (1), 103194. 10.1016/j.asej.2024.103194

[B10] Gayathri K. B. Roy S. Jyothish K. Sahaya Vijay J. Ponnusamy C. S. Augustine T. (2025). Machine learning approach on degree-sum based topological indices of bottom-up-synthesized graphene nanoribbons. Nano, 2650008. 10.1142/s1793292026500086

[B11] Gutman I. (2006). Chemical graph theory-the mathematical connection. Adv. Quantum Chem. 51, 125–138. 10.1016/S0065-3276(06)51003-2

[B12] Gutman I. (2013). Degree-based topological indices. Croat. Chemica Acta 86 (4), 351–361. 10.5562/cca2294

[B13] Gutman I. (2021). Geometric approach to degree-based topological indices: sombor indices, MATCH commun. Math. Comput. Chem. 86 (1), 11–16.

[B14] Hayat S. Imran M. Liu J. B. (2019). Correlation between the estrada index and π-electronic energies for benzenoid hydrocarbons with applications to boron nanotubes. Int. J. Quantum Chem. 119 (23), 26016. 10.1002/qua.26016

[B15] Hosoya H. Hosoi K. (1976). Topological index as applied to π-electronic systems. III. Mathematical relations among various bond orders. J. Chem. Phys. 64 (3), 1065–1073. 10.1063/1.432316

[B16] Jeyaraj S. V. Roy S. (2023). A study on efficient technique for generating vertex-based topological characterization of boric acid 2D structure. ACS Omega 8 (25), 23089–23097. 10.1021/acsomega.3c02477 37396267 PMC10308567

[B17] Jordan M. I. Mitchell T. M. (2015). Machine learning: trends, perspectives, and prospects. Science 349 (6245), 255–260. 10.1126/science.aaa8415 26185243

[B18] Jordan R. S. Wang Y. McCurdy R. D. Yeung M. T. Marsh K. L. Khan S. I. (2016). Synthesis of graphene nanoribbons *via* the topochemical polymerization and subsequent aromatization of a diacetylene precursor. Chem 1 (1), 78–90. 10.1016/j.chempr.2016.06.010

[B19] Jyothish K. Roy S. (2024). Topological characterization of [n]-triangulenes through degree-based molecular descriptors with the prediction to π-electron energy. Phys. Scr. 100 (1), 015266. 10.1088/1402-4896/ad9cd1

[B20] Jyothish K. Santiago R. Govardhan S. Hayat S. (2024). Structure-property modeling of physicochemical properties of fractal trigonal triphenylenoids by means of novel degree-based topological indices. Eur. Phys. J. E 47 (6), 42. 10.1140/epje/s10189-024-00438-3 38890172

[B21] Karthikeyan A. Priyakumar U. D. (2022). Artificial intelligence: machine learning for chemical sciences. J. Chem. Sci. 134, 1–20. 10.1007/s12039-021-01995-2 34955617 PMC8691161

[B22] Kumar C. V. Pattammattel A. (2017). Discovery of graphene and beyond, introduction to graphene, 1–15.

[B23] Liu J. Li B. W. Tan Y. Z. Giannakopoulos A. Sanchez-Sanchez C. Beljonne D. (2015). Toward cove-edged low band gap graphene nanoribbons. J. Am. Chem. Soc. 137 (18), 6097–6103. 10.1021/jacs.5b03017 25909566 PMC4456008

[B24] Lu G. Yu K. Wen Z. Chen J. (2013). Semiconducting graphene: converting graphene from semimetal to semiconductor. Nanoscale 5 (4), 1353–1368. 10.1039/c2nr32453a 23318353

[B25] Nguyen B. H. Nguyen V. H. (2016). Promising applications of graphene and graphene-based nanostructures. Adv. Nat. Sci. Nanosci. Nanotechnol. 7 (2), 023002. 10.1088/2043-6262/7/2/023002

[B26] Raihan A. (2023). A comprehensive review of artificial intelligence and machine learning applications in energy sector. J. Technol. Innovations Energy 2 (4), 1–26. 10.56556/jtie.v2i4.608

[B27] Randić M. (1975). Characterization of molecular branching. J. Am. Chem. Soc. 97 (23), 6609–6615. 10.1021/ja00856a001

[B28] Sarker I. H. (2021). Machine learning: Algorithms, real-world applications and research directions. SN Computer Science 2 (3), 160. 10.1007/s42979-021-00592-x PMC7983091 33778771

[B29] Shin S. R. Li Y. C. Jang H. L. Khoshakhlagh P. Akbari M. Nasajpour A. (2016). Graphene-based materials for tissue engineering. Adv. Drug Delivery Reviews 105, 255–274. 10.1016/j.addr.2016.03.007 27037064 PMC5039063

[B30] Son Y. W. Cohen M. L. Louie S. G. (2006). Energy gaps in graphene nanoribbons. Phys. Review Letters 97 (21), 216803. 10.1103/PhysRevLett.97.216803 17155765

[B31] Sorokin P. B. Chernozatonskii L. A. (2013). Graphene-based semiconductor nanostructures. Physics-Uspekhi 56 (2), 105–122. 10.3367/ufne.0183.201302a.0113

[B32] Sur U. K. (2012). Graphene: a rising star on the horizon of materials science. Int. J. Electrochemistry (1), 237689. 10.1155/2012/237689

[B33] Tiwari S. K. Sahoo S. Wang N. Huczko A. (2020). Graphene research and their outputs: status and prospect. J. Sci. Adv. Mater. Devices 5 (1), 10–29. 10.1016/j.jsamd.2020.01.006

[B34] Trinajstic N. (2018). Chemical graph theory. Taylor & Francis.

[B35] Vukičević D. Furtula B. (2009). Topological index based on the ratios of geometrical and arithmetical means of end-vertex degrees of edges. J. Mathematical Chemistry 46 (4), 1369–1376. 10.1007/s10910-009-9520-x

[B36] Wiener H. (1947). Structural determination of paraffin boiling points. J. Am. Chemical Society 69 (1), 17–20. 10.1021/ja01193a005 20291038

[B37] Yang X. Dou X. Rouhanipour A. Zhi L. Räder H. J. Müllen K. (2008). Two-dimensional graphene nanoribbons. J. Am. Chem. Soc. 130 (13), 4216–4217. 10.1021/ja710234t 18324813

[B38] Zhao S. Rondin L. Delport G. Voisin C. Beser U. Hu Y. (2017). Fluorescence from graphene nanoribbons of well-defined structure. Carbon 119, 235–240. 10.1016/j.carbon.2017.04.043

[B39] Zhou B. Trinajstić N. (2009). On a novel connectivity index. J. Mathematical Chemistry 46 (4), 1252–1270. 10.1007/s10910-008-9515-z

